# Magnetic Resonance Imaging of Transplanted Porcine Neonatal Pancreatic Cell Clusters Labeled with Exendin-4-Conjugated Manganese Magnetism-Engineered Iron Oxide Nanoparticles

**DOI:** 10.3390/nano12071222

**Published:** 2022-04-05

**Authors:** Jyuhn-Huarng Juang, Jiun-Jie Wang, Chia-Rui Shen, Sung-Han Lin, Chen-Yi Chen, Chen-Wei Kao, Chen-Ling Chen, Shu-Ting Wu, Zei-Tsan Tsai, Yun-Ming Wang

**Affiliations:** 1 Division of Endocrinology and Metabolism, Department of Internal Medicine and Center for Tissue Engineering, Chang Gung Memorial Hospital, Taoyuan 33305, Taiwan; je3474@gmail.com (C.-Y.C.); lian8807111@gmail.com (C.-W.K.); jenny74513@gmail.com (C.-L.C.); 2 Department of Medicine, College of Medicine, Chang Gung University, Taoyuan 33302, Taiwan; 3 Department of Medical Imaging and Radiological Sciences, College of Medicine, Chang Gung University, Taoyuan 33302, Taiwan; jiunjie.wang@gmail.com (J.-J.W.); image.lin@gmail.com (S.-H.L.); 4 Department of Diagnostic Radiology, Chang Gung Memorial Hospital, Keelung 20401, Taiwan; 5 Department of Medical Biotechnology and Laboratory Science, College of Medicine, Chang Gung University, Taoyuan 33302, Taiwan; crshen@mail.cgu.edu.tw (C.-R.S.); proteinwhite@livemail.tw (S.-T.W.); 6 Department of Ophthalmology, Chang Gung Memorial Hospital, Taoyuan 33305, Taiwan; 7 Molecular Imaging Center, Chang Gung Memorial Hospital, Taoyuan 33305, Taiwan; zeitsan@ms9.hinet.net; 8 Department of Biological Science and Technology, Institute of Molecular Medicine and Bioengineering, Center for Intelligent Drug Systems and Smart Bio-Devices (IDS2B), National Yang Ming Chiao Tung University, Hsinchu 300, Taiwan

**Keywords:** porcine neonatal pancreatic cell clusters, transplantation, exendin-4-conjugated manganese magnetism-engineered iron oxide nanoparticles, magnetic resonance imaging

## Abstract

Recently, we have shown that manganese magnetism-engineered iron oxide nanoparticles (MnMEIO NPs) conjugated with exendin-4 (Ex4) act as a contrast agent that directly trace implanted mouse islet β-cells by magnetic resonance imaging (MRI). Here we further advanced this technology to track implanted porcine neonatal pancreatic cell clusters (NPCCs) containing ducts, endocrine, and exocrine cells. NPCCs from one-day-old neonatal pigs were isolated, cultured for three days, and then incubated overnight with MnMEIO-Ex4 NPs. Binding of NPCCs and MnMEIO-Ex4 NPs was confirmed with Prussian blue staining in vitro prior to the transplantation of 2000 MnMEIO-Ex4 NP-labeled NPCCs beneath the left renal capsule of six nondiabetic nude mice. The 7.0 T MRI on recipients revealed persistent hypointense areas at implantation sites for up to 54 days. The MR signal intensity of the graft on left kidney reduced 62–88% compared to the mirror areas on the contralateral kidney. Histological studies showed colocalization of insulin/iron and SOX9/iron staining in NPCC grafts, indicating that MnMEIO-Ex4 NPs were taken up by mature β-cells and pancreatic progenitors. We conclude that MnMEIO-Ex4 NPs are excellent contrast agents for detecting and long-term monitoring implanted NPCCs by MRI.

## 1. Introduction

Human islet transplantation has cured people with type 1 diabetes. However, multiple transplants are often required to achieve insulin independence [1,2,3]. In order to solve the problem of limited supply of donor pancreas, alternative β-cell sources have been explored, especially xenogeneic islets and pluripotent stem cells [4,5]. The pig is a potential source of xenogeneic islets, but adult pig islets are difficult to isolate [6], and fetal porcine islets do not respond to glucose stimulation [6,7]. In contrast, porcine neonatal pancreatic cell clusters (NPCCs) are easily isolated, capable of secreting insulin after glucose load, and restore euglycemia posttransplantation in diabetic mice [8,9,10,11,12], pigs [13], and nonhuman primates [14]. Nevertheless, they are immature and continuously differentiate in vitro [8,9,15] and in vivo [9,10,15].

A precise, reproducible non-invasive imaging is critical for evaluation of islet engraftment and early detection of islet loss [16,17]. Magnetic resonance imaging (MRI) was utilized to track islet grafts labeled with dextran-coated superparamagnetic iron oxide (SPIO), including Ferucarbotran (Resovist^®^) and ferumoxide (Feridex^®^, Endorem^TM^) in mice [18,19,20,21], rats [22,23,24,25,26,27,28,29], baboons [30], and humans [31,32]. However, Resovist^®^ and Feridex^®^ were withdrawn due to lack of clinical data of their efficacy, specificity, and benefits [33]. This calls for a desire need in the development of new MR contrast agents for islet imaging. Chitosan, one of many natural polysaccharides, contains primary amines which is benefit for metal ion chelation and nanoparticle immobilization [34,35]. Therefore, we had developed SPIO nanoparticles coated with chitosan (CSPIO NPs) [36,37] that were safe and effective in long-term imaging transplanted MIN6 β-cells [38], NPCCs [39], and mouse islet iso- [40,41] and allo-grafts [41,42]. Since SPIO NPs may be taken up by a variety of cells in islets [19,25,41], those MR images are not specific for β-cells. Therefore, searching β-cell targeted MRI probes is needed for imaging transplanted β-cells.

Manganese ion shortens the T1 and T2 relaxation time of neighboring water protons [43] and is a potential contrast agent for MRI. Magnetism-engineered iron oxide (MEIO) NPs is a novel class of iron oxide NPs which possess high and tunable nanomagnetism [44]. The addition of manganese, MnMEIO NPs, further enhances MR signal. We then fabricated nanoparticles that consist of a copolymer shell of silane, MnMEIO core and amine-functionalized poly(ethylene glycol) (PEG) [45,46]. The flexible PEG arms reduce non-specific binding of MnMEIO-silane-NH_2_-mPEG NPs to cells by shielding positive charges of non-conjugated reactive amine groups. Furthermore, we demonstrated that specific and effective targeting of mouse epidermal growth factor receptor (EGFR)-expressing tumors could be achieved by conjugating reactive amine groups on MnMEIO-silane-NH_2_-mPEG NPs with EGFR antibody [46]. The glucagon-like peptide-1 (GLP-1) receptor is a specific surface marker of pancreatic islet β-cells and is not found in murine and human islet α-, δ- and PP-cells [47]. Studies have shown that exendin-4 (Ex4), a GLP-1 analog, can be used as β-cell-specific probes for in vivo MR imaging of implanted insulinoma [48] and native pancreatic islets in mice [49,50]. Following this strategy, we conjugated MnMEIO NPs with Ex4 (MnMEIO-Ex4 NPs) as a β-cell-specific MRI probe and confirmed that MnMEIO-Ex4 NPs-labeled mouse β-cells could be detected and traced by MRI after transplantation [51]. As we know, NPCCs are different from mature adult cells since they replicate and differentiate post transplant [8,9,10,11,12,15]. Therefore, in the present study, we further investigated whether or not MnMEIO-Ex4 NPs could be used in imaging NPCC grafts by MRI.

## 2. Materials and Methods

### 2.1. Materials

Collagenase type V, exendin-4 (Ex4), manganese (II) chloride (MnCl_2_·4H_2_O, 99%), iron (III) acetylacetonate (Fe(acac)_3_, 99.9%), methyl poly(ethylene glycol) (mPEG, M.W. = 2000), *N*-hydroxysuccinimide (NHS), *N*-ethyl-*N*′-(3-dimethylaminopropyl) carbodiimide (EDC), oleic acid (90%), oleylamine (90%), osmium tetroxide (1%) and Prussian blue were from Sigma–Aldrich (St. Louis, MO, USA). Acryloyl chloride (96%) and *N*-Boc-ethylenediamine (98%) were from Alfa Aesar (Ward Hill, MA, USA). Benzyl ether and (3-aminopropyl) triethoxy silane (APTES, 98%) were from Fluka (Buchs, SG, Switzerland). *N*-Hydroxybenzotriazole (HOBt) and (benzotriazol-1-yloxy) tripyrrolidinophosphonium hexafluorophosphate (PyBOP) were from NovaBiochem (Beeston, NTH, UK). RPMI-1640 medium was from GIBCO BRL (Grand Island, NY, USA). Polyethylene (PE-50) tubing was from Clay Adams (Parsippany, NJ, USA). Guinea pig anti-swine insulin antibody was from Dako (Carpinteria, CA, USA). Rabbit polyclonal anti-SOX9 antibody (E-9) was from EMD Millipore Corporation (Temecura, CA, USA).

### 2.2. Synthesis of MnMEIO and MnMEIO-Ex4 NPs

**Figure 1:**
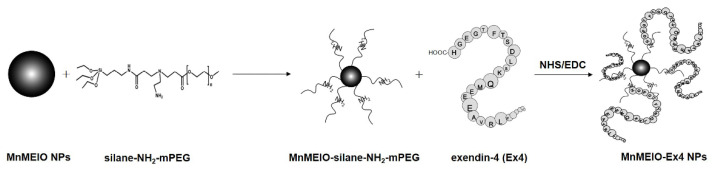
Schematic illustration of the synthesis of MnMEIO and MnMEIO-Ex4 NPs.

The synthesis of MnMEIO-silane-NH_2_-mPEG NPs has been described previously [49,50]. For MnMEIOs-Ex4 NPs preparation, 300 μL of 1.6 mg/mL EDC and 300 μL of 1 mg/mL NHS were added to 20 μL of 1 mg/mL Ex4 solution containing MnMEIO-silane-NH2-mPEG NPs (Figure 1). Based on our previous safety study, MnMEIO-Ex4 NPs with the concentration of 40 µg/mL were used for in vitro and in vivo experiments [51].

### 2.3. Animals

Male and female one-day-old pigs were obtained from a local slaughterhouse. Eight to twelve-week-old male athymic nude Balb/c mice from the National Laboratory Animal Center (Taipei, Taiwan) were used as recipients of NPCCs. All animal experiments were approved by the Institutional Animal Care and Use Committee of Chang Gung Memorial Hospital.

### 2.4. Preparation, Culture and Labeling of NPCCs

Neonatal pig pancreases were cut into fragments of ~1 to 2 mm^3^ and then digested by collagenase type V in a shaking water bath at 37 °C. The digest was cultured in RPMI-1640 medium at 37 °C (5% CO_2_, 95% air) in humidified air [12,15,39] for three days. NPCCs were then incubated overnight with MnMEIO-Ex4 NPs before in vitro studies and transplantation.

### 2.5. Binding of MnMEIO-Ex4 NPs by NPCCs

NPCCs were incubated overnight with MnMEIO-Ex4 NPs and then the binding of MnMEIO-Ex4 NPs was examined by Prussian blue staining. After fixation in 4 vol% formaldehyde solution for 30 min, NPCCs were stained for the presence of iron with Prussian blue, freshly prepared potassium ferrocyanate solution (mixture of equal volume of 4 wt% potassium ferrocyanate with 4 vol% hydrochloric acid) for 30 min. Cells with blue particles were considered bound [51].

### 2.6. Transplantation of MnMEIO-Ex4 NPs-Labeled NPCCs

Two thousand NPCCs labeled with MnMEIO-Ex4 NPs were implanted beneath the left renal capsule of each of six nondiabetic nude mouse. NPCCs were carefully transferred into a PE-50 tubing connected to a 200-µL pipette tip prior to centrifugation. Capsulotomy at the lower pole of the left kidney was performed. The tip of the tubing was then inserted and advanced under the capsule towards the injection site [12,15,39].

### 2.7. In Vivo MR Scanning

After transplantation, both transversal and coronal MR images were acquired from a 7.0 T MRI scanner (Clinscan, Bruker, Ettlingen, Germany) in six recipients using a T2-weighted turbo spin-echo sequence with a surface coil. The imaging parameters are: TR = 4532/3700 ms; TE = 37 ms; FOV = 32 × 55 mm; matrix size = 266 × 448; slice thickness = 0.5 mm. MR signal intensity of the graft at the left kidney and the mirror area at the contralateral kidney, a within-subject control, was calculated [37,38,39,40,41,42,51].

### 2.8. Histological Study of MnMEIO-Ex4 NPs-Labeled NPCC Grafts

NPCC grafts were retrieved from six recipients, two at day fifteen, fifty-one, and fifty-five after implantation, respectively. The graft was fixed in a formalin solution, embedded in paraffin and sectioned. Sections were then stained for β-cells with a guinea pig anti-swine insulin antibody, pancreatic progenitors with a rabbit polyclonal anti-SOX9 antibody, and for iron with Prussian blue [15,38,39,40,41,42,51].

### 2.9. Statistical Analysis

The MR signal intensity was computed as mean and standard deviation. All statistics were analyzed by PASW Statistics 21 (IBM Corp. Released 2012. IBM SPSS Statistics for Windows. Armonk, NY, USA: IBM Corp.). For paired comparisons of mean values of the graft at the left kidney and the mirror area at the contralateral kidney, we first checked the normality of the distribution of the variable by using the Kolmogorov-Smirnov test. If both samples passed the normality test, the independent t-test was performed. The Mann-Whitney U test (Wilcoxon test) was carried out if any one sample failed with the normality test. The *p*-value less than 0.05 was considered statistically significant.

## 3. Results

### 3.1. Binding of MnMEIO-Ex4 NPs to NPCCs

**Figure 2:**
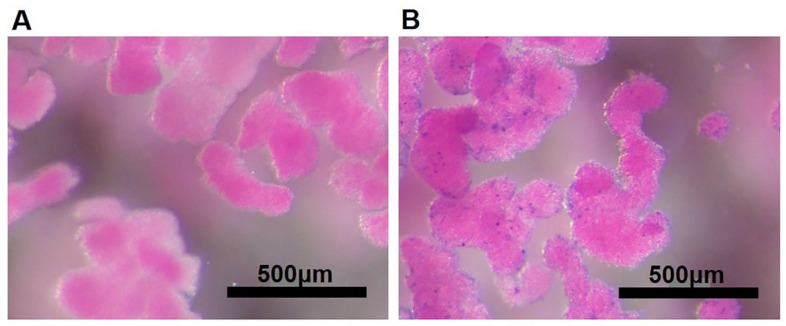
Binding of MnMEIO-Ex4 NPs to NPCCs. NPCCs were incubated overnight without (**A**) or with (**B**) MnMEIO-Ex4 NPs. The iron stained by Prussian blue expressed blue color only on the cell surface of NPCCs with MnMEIO-Ex4 NPs loading.

We have developed and characterized MnMEIO-Ex4 NPs, a novel MR contrast agent, with a z-average diameter of 70.2 ± 2.3 nm, a zeta potential of 0.6 ± 0.1 mV, a polydispersity index (PDI) of 0.36 ± 0.01 and an iron concentration of 0.43 mg/mL [51]. To examine cellular binding of MnMEIO-Ex4 NPs, NPCCs were first incubated overnight with MnMEIO-Ex4 NPs and then stained with Prussian blue. We found there was no blue staining on the surface of NPCCs without MnMEIO-Ex4 NPs loading (Figure 2A) while the blue spots were located on all MnMEIO-Ex4 NPs-loaded NPCCs (Figure 2B), indicating the binding of MnMEIO-Ex4 NPs to NPCCs.

### 3.2. In Vivo MR Images of MnMEIO-Ex4 NPs-Labeled NPCC Grafts

**Figure 3:**
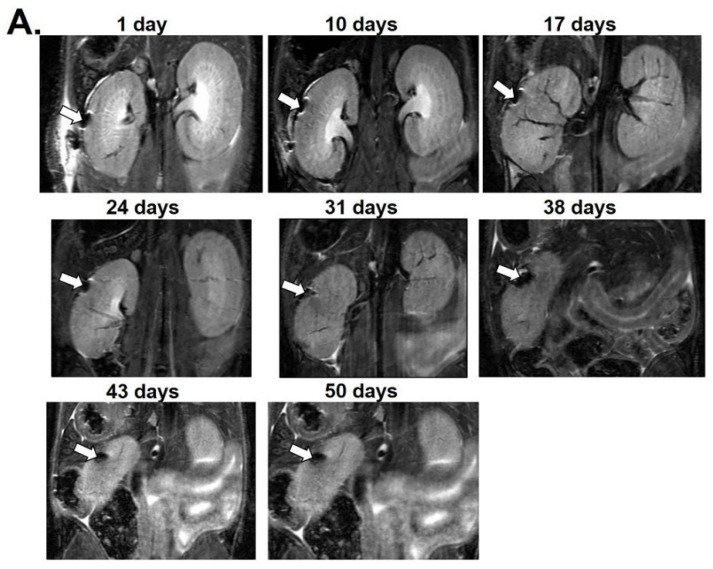

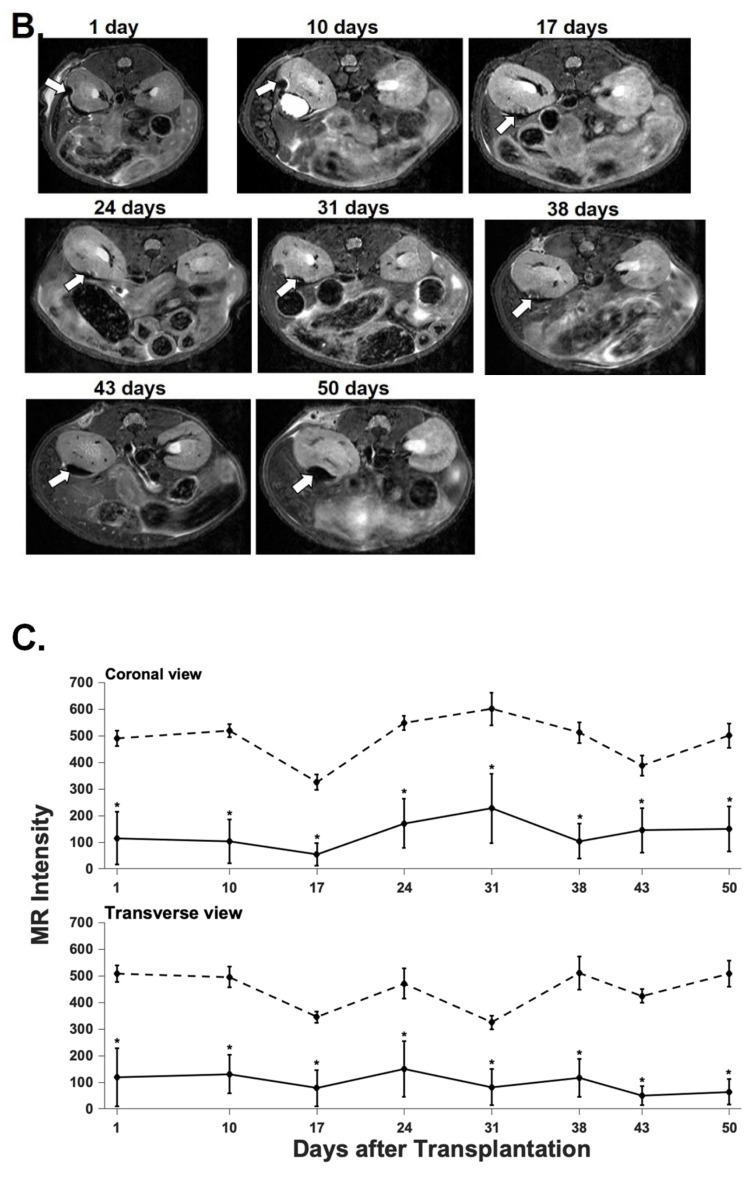
In vivo magnetic resonance (MR) images of a MnMEIO-Ex4-labeled NPCCs graft followed-up for 50 days posttransplantation. Two thousand MnMEIO-Ex4-labeled NPCCs were transplanted under the left kidney capsule of a nude mouse. The recipient was scanned by a 7.0 T MRI machine with coronal (**A**) and transverse (**B**) sections. The graft was indicated by arrows. (**C**) The time course of the MR signal intensity of the graft on left kidney (solid line) and the mirror area on the contralateral kidney (dash line) in the mouse. * *p* < 0.001.

**Figure 4:**
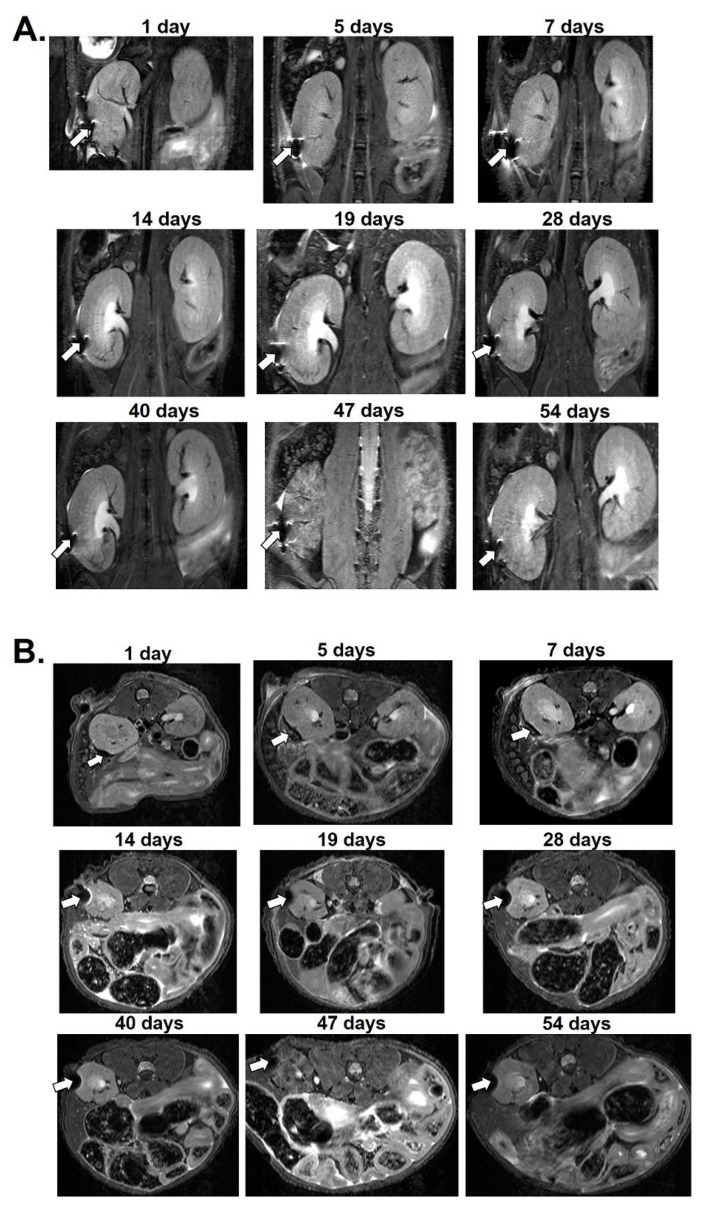

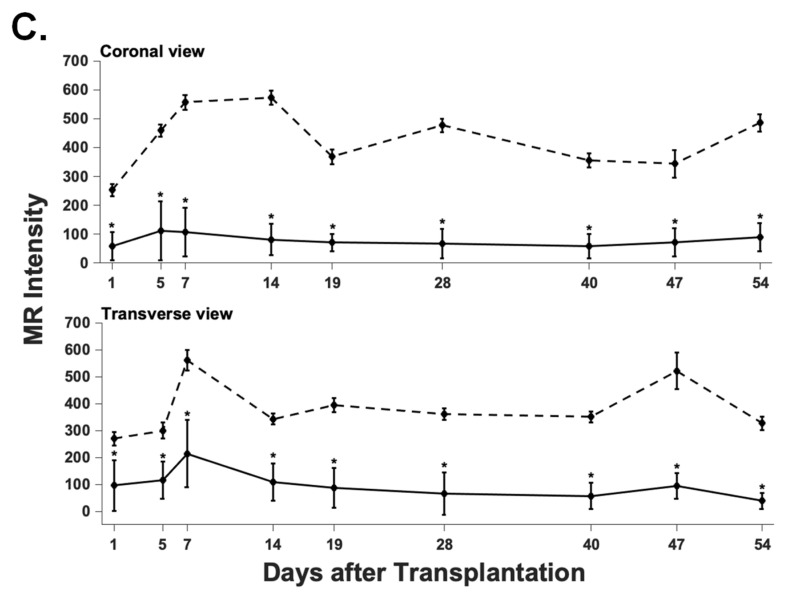
In vivo magnetic resonance (MR) images of a MnMEIO-Ex4-labeled NPCCs graft followed-up for 54 days posttransplantation. Two thousand MnMEIO-Ex4-labeled NPCCs were transplanted under the left kidney capsule of a nude mouse. The recipient was scanned by a 7.0 T MRI machine with coronal (**A**) and transverse (**B**) sections. The graft was indicated by arrows. (**C**) The time course of the MR signal intensity of the graft on left kidney (solid line) ae mirror area on the contralateral kidney (dash line) in the mouse. * *p* < 0.001.

For in vivo MRI, 2000 MnMEIO-Ex4 NPs-labeled NPCCs were transplanted under the left kidney capsule of six nude mice. After transplantation, these mice were scanned by a 7.0 T MRI machine at various time points for 8, 50 (Figure 3) and 54 (Figure 4) days. The MR images of the MnMEIO-Ex4 NPs-labeled NPCC graft revealed persistent hypointense areas located at the site of implantation (indicated by arrows in Figure 3A,B and Figure 4A,B). The quantitative analysis showed a significant (62–88%) reduction of the MR signal intensity in the graft on left kidney when compared to the mirror area on the contralateral kidney at all time points (*p* < 0.001) (Figure 3C and Figure 4C). This indicates that MnMEIO-Ex4 NPs can be applied in tracing NPCCs grafts for a long period of time.

### 3.3. Histological Studies of MnMEIO-Ex4 NPs-Labeled NPCC Grafts

**Figure 5:**
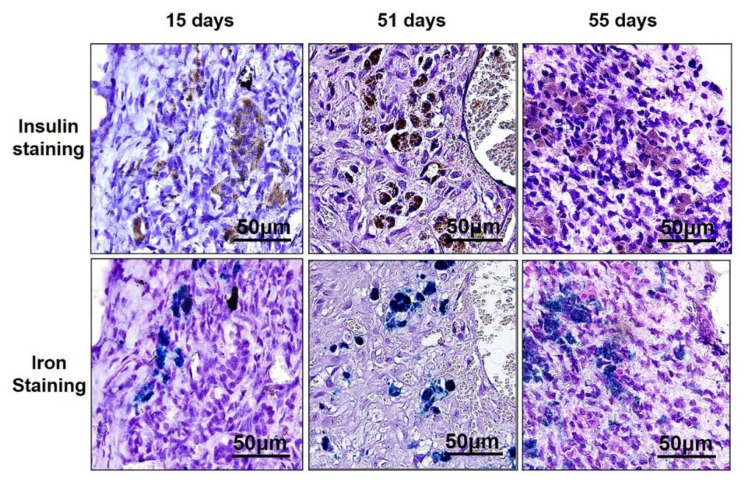
Insulin (brown color in upper panel) and Prussian blue (blue color in lower panel) staining of MnMEIO-Ex4 NP-labeled NPCC grafts removed at day 15, 51, and 55 post transplantation.

**Figure 6:**
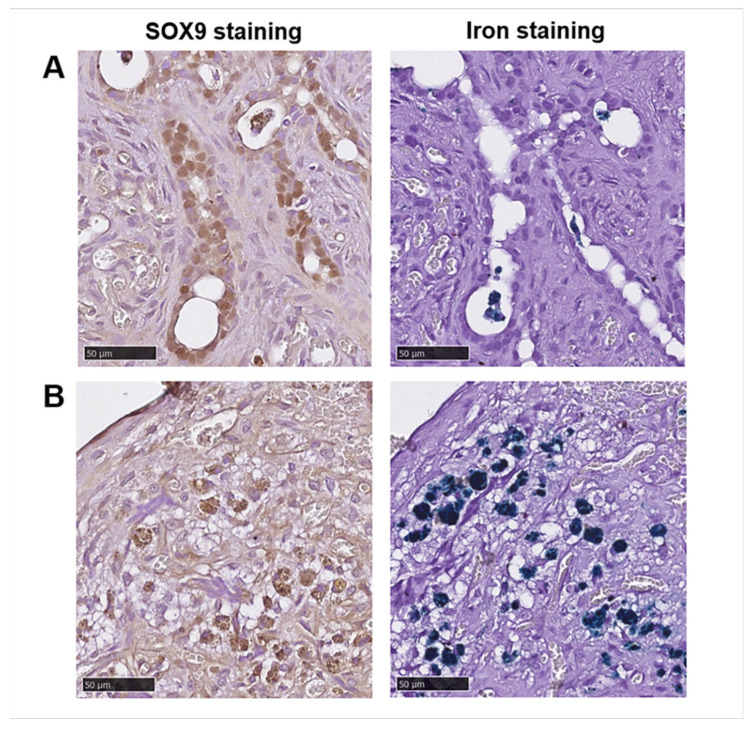
SOX9 and Prussian blue staining of MnMEIO-Ex4 NP-labeled NPCC grafts removed at 51 days after transplantation. (**A**) SOX9-positive cells (left panel, brown color) around ducts were not stained with iron (right panel). (**B**) Cells with colocalization of SOX9 (left panel, brown color) and iron (right panel, blue color).

MnMEIO-Ex4 NPs-labeled NPCC grafts were removed from six recipients, two at day 15, 51, and 55 post transplantation, respectively. To examine the graft histology, anti-insulin and anti-SOX9 antibodies as well as Prussian stain were used to stain pancreatic β-cells, pancreatic progenitors and iron, respectively. As shown in Figure 5, insulin (upper panel) and iron (lower panel) staining were positive and colocalized in 15-, 51-, and 55-day grafts. We found abundant SOX9-positive cells in (Figure 6A) and outside (Figure 6B) pancreatic ducts of the 51-day graft. However, colocalization of SOX9 and Prussian blue staining was only observed in those outside pancreatic ducts (Figure 6B).

## 4. Discussion

Previously, we coated SPIO NPs with chitosan [36,37] and successfully imaged CSPIO NPs-labeled NPCC grafts by MRI [39]. Since SPIO NPs are taken up through endocytosis by cells [19,25,41], those MR images are not necessarily representative of β-cells. To specifically image transplanted β-cells, we conjugated an MR contrast agent MnMEIO NPs with GLP-1 analog Ex4 which can bind GLP-1 receptors on the surface of β-cells. Our results showed that MnMEIO NPs were taken up by β-cells through receptor-mediated endocytosis and MnMEIO-Ex4 NPs were safe and effective for the detection and long-term tracing of transplanted mouse islet β-cells by MRI [51]. In this study, we further demonstrated that MnMEIO-Ex4 NPs could bind NPCCs and MnMEIO-Ex4 NPs-labeled NPCC grafts could be visualized and monitored by MRI for a long period of time.

For in vivo MR imaging, we transplanted 2000 MnMEIO-Ex4 NPs-labeled NPCCs beneath the left renal capsule in each nude mouse. During the 50- and 54-day follow-up, there was a reduction in the MR signal intensity of the graft on the left kidney by 62–88% compared to the mirror areas on the contralateral kidney. These findings are consistent with our previous observation with CSPIO NPs-labeled NPCC grafts which showed that 60–80% reduction of the MR signal intensity [39]. This indicates that MnMEIO-Ex4 NPs are as effective as CSPIO NPs in detecting NPCC grafts.

Through GLP-1 receptors, MnMEIO-Ex4 NPs can be taken up by β-cells (i.e., receptor-mediated endocytosis) [51]. Therefore, the labeled cells in NPCC grafts showed hypointense areas on in vivo MR images. This notion is confirmed by our histological studies of colocalization of insulin/iron and SOX9/iron staining in NPCC grafts. In fact, NPCCs are clusters of pancreatic cells containing ducts, endocrine and exocrine cells [8,9,15]. In mouse, rat, and human pancreases, GLP-1 receptors express not only on islet β-cells but also in duct tissues [47,52,53] where progenitor cells are located. In NPCC graft, we found abundant cells stained positive for insulin and SOX9, indicating the existence of mature β-cells and pancreatic progenitors. We only observed the colocalization of the SOX9 and Prussian blue staining in cells outside but not in pancreatic ducts. Presumably, SOX9-positive cells in pancreatic ducts are newly formed progenitors [54] which were not present during the labeling of MnMEIO-Ex4 NPs before transplantation. That’s why we did not find colocalization of SOX9 and Prussian blue staining in those cells. Taken together, MnMEIO-Ex4 NPs were taken up by NPCCs with GLP-1 receptors (i.e., mature β-cells and pancreatic progenitors), and these cells showed positive MR images. In this regard, MnMEIO-Ex4 NPs are superior to CSPIO NPs. To the best of our knowledge, we are the first to apply GLP-1 receptor probes in imaging NPCC grafts.

## 5. Conclusions

In addition to MnMEIO-Ex4 NPs-labeled mouse islet isografts [49], in this study, we further extended the application of MnMEIO-Ex4 NPs in tracing NPCCs by MRI. Our results showed that MnMEIO-Ex4 NPs bound NPCCs in vitro and NPCCs grafts revealed persistent positive MR images for up to 54 days after transplantation. Histological studies also confirmed colocalization of the insulin/iron and SOX9/iron staining in NPCC grafts. We conclude that MnMEIO-Ex4 NPs are excellent contrast agents for detecting and long-term monitoring transplanted NPCCs.

## Data Availability

Not applicable.
